# The complete chloroplast genome of *Narcissus poeticus* L. (Amaryllidaceae: Amaryllidoideae)

**DOI:** 10.1080/23802359.2018.1521311

**Published:** 2018-10-08

**Authors:** Kálmán Könyves, Jordan Bilsborrow, John David, Alastair Culham

**Affiliations:** a Royal Horticultural Society Garden Wisley, Woking, United Kingdom;; b Herbarium, School of Biological Sciences, University of Reading, Reading, United Kingdom

**Keywords:** Narcissus, Amaryllidaceae, plastome, ycf1, cemA

## Abstract

The first complete chloroplast genome sequence for *Narcissus* is assembled and annotated in this study. The total length of the *N. poeticus* chloroplast genome is 160,099 bp and comprises the large single copy (LSC) spanning 86,445 bp, the small single copy (SSC) spanning 16,434 bp, and two inverted repeat regions each of 28,610 bp length. The truncated copy of *ycf1* before the junction between IR_B_ and SSC was 1277–2428 bp longer than in other included Asparagales samples. A potential pseudogene, *cemA*, was also identified. This is the first reported plastome for Amaryllidaceae subfamily Amaryllidoideae.

*Narcissus* L. is a member of Amaryllidaceae, a family comprising several horticulturally important plant genera (Heywood et al. 2007). *Narcissus poeticus* is the type species of the genus, however, its placement among other daffodils remains unresolved (Marques et al. 2017). Currently, there are no published daffodil chloroplast genomes and only a few genomes are available for Amaryllidaceae, none for subfamily Amaryllidoideae. Here we report the complete chloroplast genome sequence of *N. poeticus* to address this and to identify new regions of genomic variability.

Leaf material was collected in silica gel from *N. poeticus* grown at RHS Garden Wisley, UK (51.312695° N, 0.476724° W). Herbarium voucher specimen was deposited at WSY (WSY0108940). Total genomic DNA was extracted using the QIAGEN DNeasy Plant Mini Kit (QIAGEN, Manchester, UK). Library development and 150 bp PE sequencing on 1/16 of an Illumina HiSeq 4000 lane were done at the Oxford Genomics Centre (Oxford, UK). The chloroplast genome was assembled with Fast-Plast v1.2.6 (McKain and Wilson 2017) and NovoPlasty v2.7.0 (Dierckxsens et al. 2017). Fast-Plast assemblies were run with all 38 M reads. Reads were trimmed to remove NEB-PE adapter sequences. Bowtie reference index was built with Asparagales chloroplast genomes included in Fast-Plast. For the NovoPlasty assembly, adapters were trimmed with Trimmomatic v0.36 (Bolger et al. 2014) using the same adapter sequences. A *ndhF* sequence of *N. poeticus* (KT124416) was used as the starting seed and memory was limited to 8 Gb. All other parameters were unchanged. Junctions of the inverted repeats were confirmed by Sanger sequencing.

The complete chloroplast genome was annotated using Verdant (McKain et al. 2017) and corrected by comparing it with published annotations (*Hesperoyucca whipplei* – KX931459; *Hosta ventricosa* – KX931460; *Yucca schridigera* – KX931469; *Cocos nucifera* – KF285453). The *N. poeticus* cpDNA genome sequence was aligned with 13 published Asparagales plastome sequences using MAFFT (Katoh and Standley 2013). A maximum likelihood estimate was conducted with RAxML v8.2.11 (Stamatakis 2014) within Geneious v11.1.5 (http://www.geneious.com, Kearse et al. 2012) using model GTR + G and 1000 bootstrap replicates.

The chloroplast genome sequence of *N. poeticus* (MH706763) is 160,099 bp, comprises the large single copy (LSC) spanning 86,445 bp, the small single copy (SSC) spanning 16,434 bp, and two inverted repeat regions each of 28,610 bp length. The junction between IR_B_ and SSC (*J*_SB_) is 34 bp within the *ndhF* gene. The junction between SSC and IR_A_ (*J*_SA_) is within the *ycf1* gene, which is 5,346 bp long, of which 2737 bp lies in the inverted repeat. Therefore, there is a 2737 bp long truncated version of *ycf1* at *J*_SB_. This is 1277–2428 bp longer than the truncated copy of *ycf1* in other Asparagales sequences analyzed here, identified by IRScope (Amiryousefi et al. 2018). A poly-A region, at the start of *cemA* shifts the ORF potentially inactivating this gene. The Amaryllidaceae samples form a clade with *N. poeticus* sister to *Allium cepa* (Figure 1) topologically consistent with Seberg et al. (2012).

**Figure 1.**
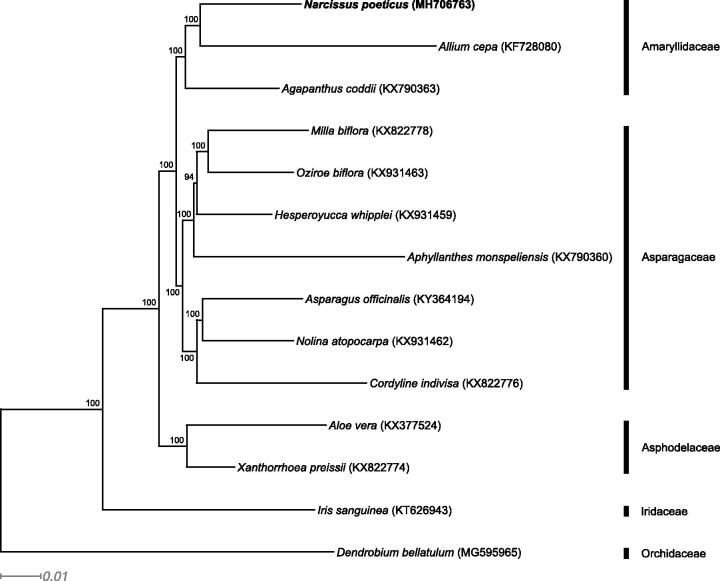
RAxML output tree with bootstrap consensus values based on 14 complete chloroplast genome sequences. The numbers at each node indicate bootstrap support. GenBank accession numbers are given in brackets. Text in bold shows the chloroplast genome developed in this study. Families of the sampled taxa are shown on the right.
